# Risk factors and nursing strategies for postoperative pain management in patients with lumbar spinal stenosis undergoing transforaminal lumbar interbody fusion: a retrospective study

**DOI:** 10.3389/fneur.2025.1646333

**Published:** 2025-09-12

**Authors:** Jingran Guo, Xiaoying Wang, Lijuan Wang, Yu Wang, Jie Li, Yi Bu

**Affiliations:** Department of Spine, The Second Hospital of Tangshan, Tangshan, China

**Keywords:** lumbar spinal stenosis, transforaminal lumbar interbody fusion, postoperative pain, risk factors, nursing strategies

## Abstract

**Objective:**

This study attempts to identify risk factors associated with postoperative pain in patients with lumbar spinal stenosis undergoing transforaminal lumbar interbody fusion (TLIF) and to propose targeted nursing strategies.

**Methods:**

We retrospectively analyzed 502 patients who underwent TLIF. Patients were grouped into mild, moderate, and severe pain groups based on postoperative pain severity. Baseline characteristics, comorbidities, sex, age, body mass index (BMI), and history of lumbar surgery were compared across groups. Preoperative serological markers such as glycated hemoglobin (HbA1c), albumin, globulin, red blood cell count (RBC), white blood cell count (WBC), platelet count (PLT), neutrophil-to-lymphocyte ratio (NLR), and C-reactive protein (CRP) were analyzed. Surgical parameters, including operative time, intraoperative blood loss, surgical segment, bone graft material, anesthesia method, drainage duration, and postoperative complications, were also assessed. Ordinal logistic regression and Extreme gradient boosting (XGBoost) models were applied to analyze risk factors influencing postoperative pain severity, with model performance assessed by Receiver Operating Characteristic (ROC) curves and calibration plots.

**Results:**

Significant differences among pain groups were observed in age, BMI, HbA1c, albumin, globulin, RBC, WBC, PLT, NLR, CRP, operative time, intraoperative blood loss, drainage duration, surgical segment, and complication rates (all *P* < 0.05). Ordinal logistic regression identified these factors as significant predictors of severe pain, with intraoperative blood loss showing the highest odds ratio (OR = 1.037, *P* < 0.001). XGBoost analysis ranked intraoperative blood loss as the top contributor. In the test set, XGBoost achieved an AUC of 0.968 compared with 0.974 for the ordinal logistic model; however, the logistic model demonstrated superior variance explanation (*R*^2^=0.728 vs. 0.710) and prediction accuracy (RMSE = 0.262 vs. 0.268; MAE = 0.116 vs. 0.146).

**Conclusion:**

Intraoperative blood loss emerged as a critical factor affecting pain severity. Both ordinal logistic regression and XGBoost models provide strong predictive performance and can effectively guide individualized nursing strategies, potentially improving postoperative recovery for TLIF patients.

## 1 Introduction

Lumbar spinal stenosis (LSS) is a common degenerative disorder characterized by narrowing of the spinal canal, lateral recess, or intervertebral foramen, resulting in compression of spinal nerves. Clinically, it manifests as lower back pain, leg weakness, and intermittent claudication, and is particularly prevalent among the elder (1). With the global trend of population aging, the incidence of LSS has been steadily increasing, creating a growing demand for effective treatment strategies (2).

For patients with severe symptoms unresponsive to conservative treatment, surgical intervention is often necessary. Among available procedures, transforaminal lumbar interbody fusion (TLIF) is widely preferred due to its minimally invasive approach, relatively low complication rates, and favorable recovery profile (3, 4). However, postoperative pain management following TLIF remains a persistent challenge. Inadequate pain control can impair functional recovery, prolong hospitalization, reduce quality of life, and increase healthcare costs (5–7).

Postoperative pain after TLIF is multifactorial, involving acute nociceptive pain from tissue injury, neuropathic pain from nerve root compression, inflammatory responses, and muscle spasms (8, 9). While pharmacologic and non-pharmacologic pain management strategies exist, there is limited evidence on patient-related and perioperative risk factors that influence postoperative pain severity, particularly within the context of nursing interventions and physiotherapy. This gap limits the ability to implement personalized pain management plans.

Therefore, this retrospective study aims to identify key risk factors influencing postoperative pain in LSS patients undergoing TLIF and to propose targeted nursing strategies for improved pain control. We hypothesize that specific preoperative, intraoperative, and postoperative factors significantly affect postoperative pain severity, and that implementing nursing strategies tailored to these risk factors can lead to better pain management outcomes in this patient population.

## 2 Materials and methods

### 2.1 Study population

This retrospective observational study was conducted at our hospital, a tertiary referral center for spinal surgery. Clinical records of patients diagnosed with LSS who underwent TLIF between January 2020 and December 2023 were reviewed. Patients were included based on the following criteria: (1) clinically diagnosed with LSS undergoing TLIF for the first time, meeting the relevant surgical indications (10); (2) complete surgical records, including preoperative examinations, intraoperative procedures, and postoperative follow-up; (3) availability of early postoperative pain assessment data at 3 days post-surgery.

Exclusion criteria included: (1) concurrent spinal conditions (e.g., scoliosis, spinal tumors); (2) history of previous lumbar surgery; (3) severe postoperative complications (e.g., infection, internal fixation failure) that could interfere with pain evaluation; (4) refusal to participate or incomplete data due to transfer to another hospital.

This study was conducted in accordance with the Declaration of Helsinki and approved by our Institutional Review Board (Approval No. 25025-57). The requirement for informed consent was waived by the ethics committee due to the retrospective nature of the study and use of anonymized data.

The sample size was estimated based on recent TLIF retrospective studies, in which intergroup differences in postoperative VAS pain scores (e.g., based on surgical levels, graft materials, or complication subgroups) typically showed effect sizes (Cohen's *d*) of 0.3 to 0.5. Assuming an expected effect size of *d* = 0.3, with a two-sided significance level (α) set at 0.05 and a statistical power (1–β) of 0.80, the minimum required sample size per group was calculated using the two-sample *t*-test formula, $n\text{=}\frac{{2(\text{Z}}_{\;\alpha \;/2}{+\text{Z}}_{\;\beta \;}{)}^{2}\;\sigma^{2}}{\;\delta^{2}}=\frac{2(1.96+0{.84)}^{2}\;\sigma^{2}}{{\left(0.3\;\sigma \;\right)}^{2}}$, yielding an estimate of ~150 cases per group. (Note: σ represents the population standard deviation and δ the expected difference; here, the standardized effect size *d* = δ/σ = 0.3 is used to simplify the calculation). A total of 502 cases were included, sufficient to support analyses involving three or more subgroups (e.g., single- vs. multilevel procedures or graft types), ensuring adequate power to detect moderate effect sizes.

### 2.2 Data collection

All data were extracted from the hospital's electronic medical record (EMR) system and surgical database by two independent researchers. Discrepancies were resolved through consensus. Pain scores were obtained from standardized nursing assessments performed by trained ward nurses using the VAS. The following information was collected: Demographic and clinical variables such as age, body mass index (BMI), and sex; Comorbidities; Preoperative serological indicators: glycated hemoglobin (HbA1c), albumin, globulin, red blood cell count (RBC), platelet count (PLT), white blood cell count (WBC), C-reactive protein (CRP), and neutrophil-to-lymphocyte ratio (NLR); Surgical parameters: operative time, intraoperative blood loss, surgical segment, bone graft material, and type of intraoperative anesthesia; Drainage tube duration; and Incidence of postoperative complications. Complications assessed included wound erythema or mild wound inflammation (without overt infection), superficial hematoma or bruising at the surgical site, transient sensory disturbances (e.g., paresthesia), mild muscle weakness not requiring intervention, delayed wound healing without dehiscence, transient urinary retention, mild postoperative nausea and vomiting, mild constipation or ileus, and transient low-grade fever without systemic infection. These complications were documented based on routine postoperative clinical assessments and nursing records, and managed conservatively without surgical re-intervention.

Postoperative pain was evaluated using the VAS (11) on the third day after surgery. Pain severity was categorized into three groups: Mild (VAS score 0–3); Moderate (VAS score 4–6); Severe (VAS score 7–10). The choice of postoperative day 3 was based on the consideration that, by this time, the residual effects of intraoperative anesthesia and immediate postoperative analgesics (including patient-controlled analgesia or epidural analgesia) had largely subsided, and most patients had initiated early mobilization under nursing supervision. This time point thus provides a more stable and clinically relevant reflection of the patient's actual pain experience during the early recovery phase.

### 2.3 Surgical procedures

All patients underwent standardized TLIF performed by experienced spine surgeons. Preoperative preparation included routine laboratory tests (e.g., complete blood count, coagulation profile, and imaging studies). Patients with comorbidities received necessary preoperative management. Under general anesthesia, patients were positioned prone. A posterolateral approach was used to expose the target intervertebral space. Under microscopic guidance, discectomy was performed, and compressive bone and ligament tissue were removed. An appropriately sized interbody fusion cage was implanted and packed with autologous bone. A spinal internal fixation system, including pedicle screws and connecting rods, was applied to ensure spinal stability. A surgical drain was placed before wound closure. Postoperative care was administered by the same nursing team. Prophylactic antibiotics were routinely used to prevent infection. The drainage tube was typically removed after 24 h, and early ambulation was encouraged based on the patient's condition. Pain levels and recovery status were evaluated on the third postoperative day.

### 2.4 Machine learning model

Two modeling approaches were employed to identify risk factors for postoperative pain severity: the Ordinal Logistic Regression Model, suitable for ordinal categorical dependent variables; and the Extreme Gradient Boosting (XGBoost) Model, capable of handling complex and high-dimensional data, which can further investigate potential risk factors. Data preprocessing involved standardization of continuous variables into *Z*-scores and one-hot encoding for categorical variables, with the dataset randomly divided into two parts: 80% for model training and the remaining 20% for testing purposes. Model training was performed separately for the ordinal logistic regression and XGBoost models. For the XGBoost model, hyperparameters (such as learning rate, maximum tree depth, and number of trees) were optimized using grid search and cross-validation. Both models were validated on training and testing sets, and their predictive performance was evaluated accordingly.

### 2.5 Sensitivity analysis

A sensitivity analysis re-included patients with severe postoperative complications (e.g., wound infection, poor incision healing, dural tear, cerebrospinal fluid leakage, neurological injury, hematoma, deep vein thrombosis, internal fixation failure). Both the ordinal logistic regression and the XGBoost model were re-run using the same preprocessing, hyperparameter tuning, and validation procedures as the primary analysis. Model performance metrics and effect sizes were compared with the main results.

### 2.6 Statistical analysis

Data processing and statistical analyses were performed using SPSS version 26.0 and R software. Continuous variables were tested for normality. Variables with a normal distribution were expressed as mean ± standard deviation ($\bar{x}$ ± *s*). Comparisons between two groups were conducted using the independent-samples *t*-test, while comparisons among multiple groups were analyzed using one-way analysis of variance (ANOVA). Pairwise comparisons between groups were adjusted using either the least significant difference (LSD-t) test or the Tukey test. For variables that did not follow a normal distribution, data were expressed as the median with interquartile range [M (P25, P75)], and group comparisons were carried out using either the Mann–Whitney *U* test or the Kruskal–Wallis test. Categorical variables were reported as counts and percentages, with comparisons made using the chi-square (χ^2^) test or Fisher's exact test when appropriate. Ordinal logistic regression and the XGBoost model were used to identify factors associated with postoperative pain severity. Model performance metrics included area under the receiver operating characteristic curve (AUC), coefficient of determination (*R*^2^), root mean square error (RMSE), and mean absolute error (MAE). A *P*-value of < 0.05 was deemed statistically significant.

### 2.7 Additional reporting statement

This study adhered to the Strengthening the Reporting of Observational Studies in Epidemiology (STROBE) (12) reporting guidelines for observational studies, and the completed checklist is provided as Supplementary material 1.

## 3 Results

### 3.1 Comparison of baseline characteristics and clinical indicators among patients with different pain levels

Analysis of patient data revealed significant differences across pain severity groups in age, BMI, HbA1c, albumin, globulin, RBC, WBC, PLT, NLR, CRP, operative time, intraoperative blood loss, surgical segment, drainage tube placement duration, and postoperative complication rate (all *P* < 0.05; Table 1).

**Table 1:** Comparison of baseline characteristics among groups.

**Variables**	**Mild pain group (*n* = 96)**	**Moderate pain group (*n* = 208)**	**Severe pain group (*n* = 198)**	***F/χ^2^***	***P***
Age (years)	52.50 ± 4.70	55.72 ± 4.927	58.01 ± 6.01	34.876	< 0.001
Sex (male/women)	55/41	133/75	110/88	3.169	0.205
BMI (kg/m^2^)	24.45 ± 2.457	26.64 ± 2.70	29.06 ± 2.79	101.856	< 0.001
History of smoking (yes/no)	25/71	55/153	45/153	0.831	0.660
History of alcohol consumption (yes/no)	20/76	48/160	30/168	4.185	0.123
HbA1c (%)	5.67 ± 0.45	5.94 ± 0.55	6.22 ± 0.658	37.357	< 0.001
Albumin (g/L)	42.34 ± 3.21	40.66 ± 3.02	39.04 ± 3.38	38.821	< 0.001
Globulin (g/L)	25.67 ± 2.34	27.80 ± 2.47	30.063 ± 2.92	97.853	< 0.001
RBC (× 10^12^/L)	4.56 ± 0.34	4.75 ± 0.46	4.92 ± 0.57	15.71	< 0.001
WBC (× 10^9^/L)	6.78 ± 1.23	7.78 ± 1.48	8.91 ± 1.47	67.953	< 0.001
PLT (× 10^9^/L)	234.56 ± 34.56	254.98 ± 46.60	275.76 ± 57.10	23.643	< 0.001
NLR	2.10 ± 0.45	2.51 ± 0.56	2.94 ± 0.70	86.368	< 0.001
CRP (mg/L)	5.67 ± 2.34	9.13 ± 3.345	12.54 ± 4.41	109.127	< 0.001
Operative time (min)	120.17 ± 19.96	139.93 ± 25.23	157.30 ± 30.52	65.335	< 0.001
Intraoperative blood loss (mL)	147.33 ± 31.06	201.58 ± 39.95	254.28 ± 49.69	213.685	< 0.001
Drainage tube placement duration (days)	2.11 ± 0.638	2.60 ± 0.69	3.00 ± 0.78	51.879	< 0.001
Surgical segment (single-segment/multi-segment)	70/26	120/88	100/98	13.312	0.001
Incidence of postoperative complications (%)	10.42 (10/96)	20.19 (42/208)	30.30 (60/198)	15.671	< 0.001

BMI, body mass index; HbA1c, glycated hemoglobin; RBC, red blood cell count; WBC, white blood cell count; PLT, platelet count; NLR, neutrophil-to-lymphocyte ratio; CRP, C-reactive protein.

### 3.2 Ordinal logistic regression analysis of risk factors for postoperative pain severity after TLIF

**Figure 1:**
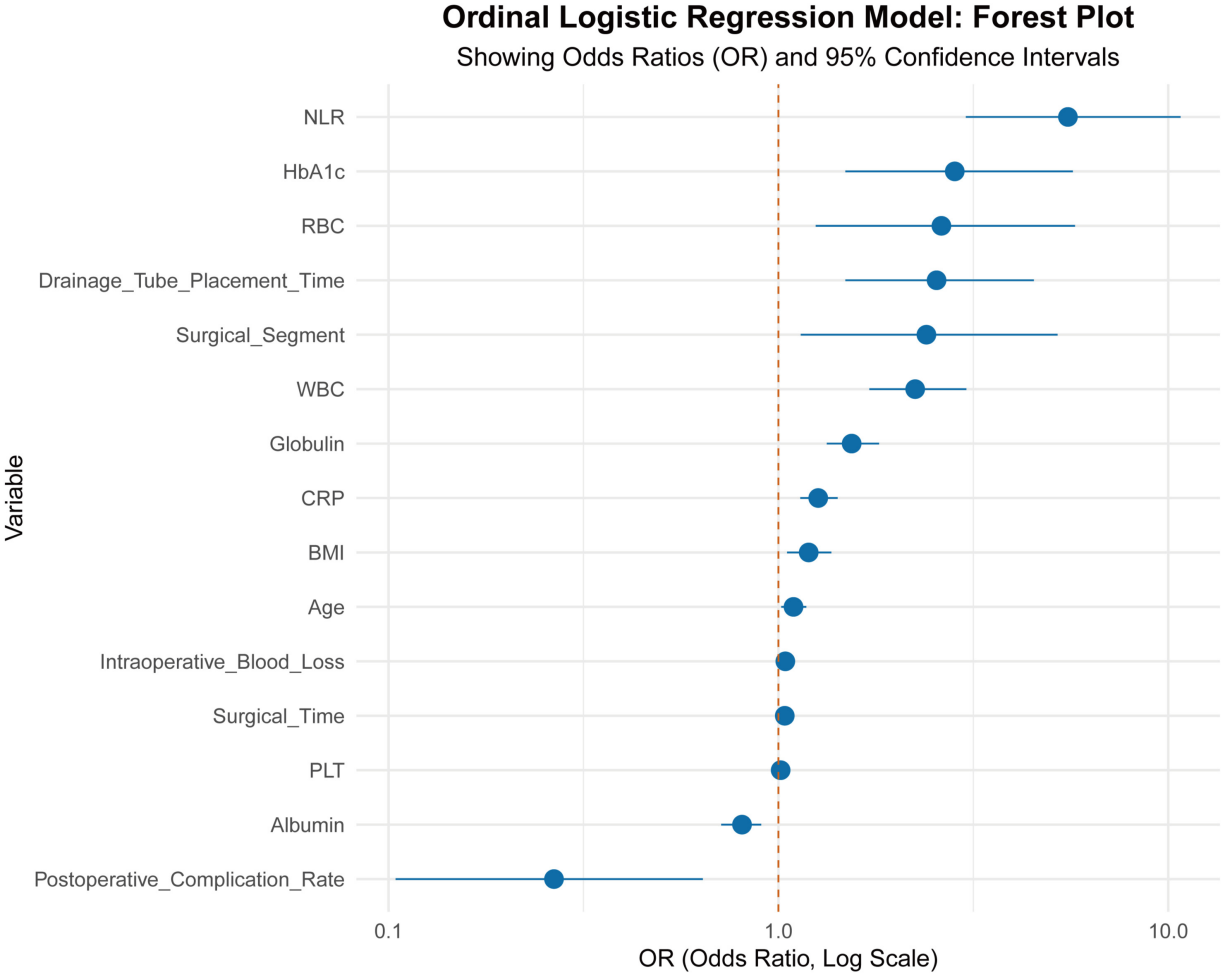
Ordinal logistic regression model: forest plot.

Prior to modeling, variance inflation factors (VIFs) were calculated to assess potential multicollinearity, with all values < 5, indicating no significant multicollinearity. An ordinal logistic regression model was constructed using variables that showed significant differences in univariate analysis. Pain severity served as the dependent variable (0 = mild pain, 1 = moderate pain, 2 = severe pain). Surgical segment and postoperative complication rate were treated as categorical variables, whereas age, BMI, HbA1c, albumin, globulin, RBC, WBC, PLT, NLR, CRP, operative time, intraoperative blood loss, and drainage tube placement time were included as covariates. The results indicated that age, BMI, HbA1c, albumin, globulin, WBC, PLT, NLR, CRP, operative time, intraoperative blood loss, drainage tube placement time, surgical segment, and postoperative complication rate were associated with an increased risk of severe postoperative pain following TLIF (*P* < 0.05). See Table 2 and Figure 1.

**Table 2:** Ordinal logistic regression analysis of risk factors associated with postoperative pain after TLIF.

**Variables**	***B***	***S_*b*_***	***Waldx^2^***	***P***	***OR***
Age	0.08	0.031	6.677	0.010	1.083
BMI	0.209	0.057	13.519	0.000	1.232
HbA1c	0.931	0.291	10.206	0.001	2.537
Albumin	−0.162	0.051	9.956	0.002	0.850
Globulin	0.379	0.065	33.471	0.000	1.461
RBC	0.586	0.313	3.505	0.061	1.797
WBC	0.648	0.118	29.921	0.000	1.912
PLT	0.012	0.003	12	0.001	1.012
NLR	1.824	0.286	40.553	0.000	6.197
CRP	0.244	0.049	24.794	0.000	1.276
Operative time	0.034	0.007	25.697	0.000	1.035
Intraoperative blood loss	0.036	0.005	61.767	0.000	1.037
Drainage tube placement time	0.903	0.233	15.035	0.000	2.467
Surgical segment	−0.778	0.326	5.701	0.017	0.459
Postoperative complication rate	1.064	0.396	7.223	0.007	2.898

BMI, body mass index; HbA1c, glycated hemoglobin; RBC, red blood cell count; WBC, white blood cell count; PLT, platelet count; NLR, neutrophil-to-lymphocyte ratio; CRP, C-reactive protein.

### 3.3 XGBoost model analysis of risk factors for postoperative pain severity after TLIF

**Figure 2:**
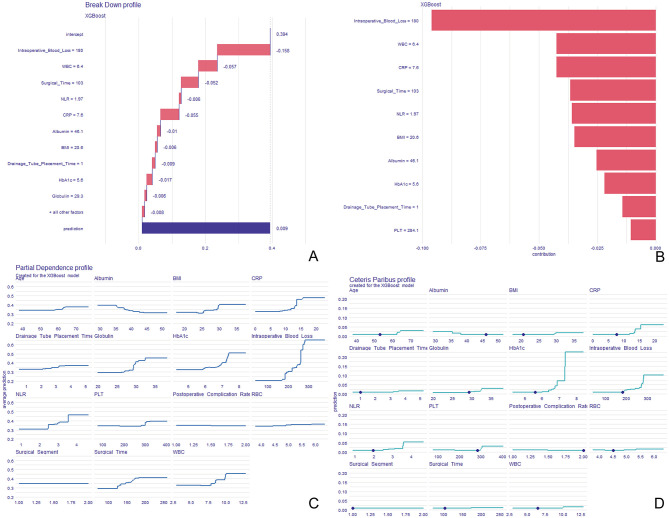
Risk factor analysis for postoperative pain levels after TLIF based on the XGBoost model. **(A)** Model explanation using breakdown analysis; **(B)** feature importance plot; **(C)** partial dependence plot; **(D)** ceteris paribus plot. TLIF, transforaminal lumbar interbody fusion; XGBoost, extreme gradient boosting.

The XGBoost model identified that age, BMI, HbA1c, albumin, globulin, WBC, PLT, NLR, CRP, operative time, intraoperative blood loss, drainage tube placement duration, surgical segment, and postoperative complication rate were significant risk factors for severe postoperative pain after TLIF. The Breakdown Profile (Figure 2A) showed a baseline prediction value of 0.394, with intraoperative blood loss contributing the most to pain severity prediction (−0.158). The feature importance plot (Figure 2B) also identified intraoperative blood loss as the most influential factor for postoperative severe pain. Moreover, Partial Dependence Profile (Figure 2C) and Ceteris Paribus Profile (Figure 2D) demonstrated that both CRP and intraoperative blood loss were critical of predicting severe pain following TLIF.

### 3.4 Comparison of the ordinal logistic regression and XGBoost models

**Figure 3:**
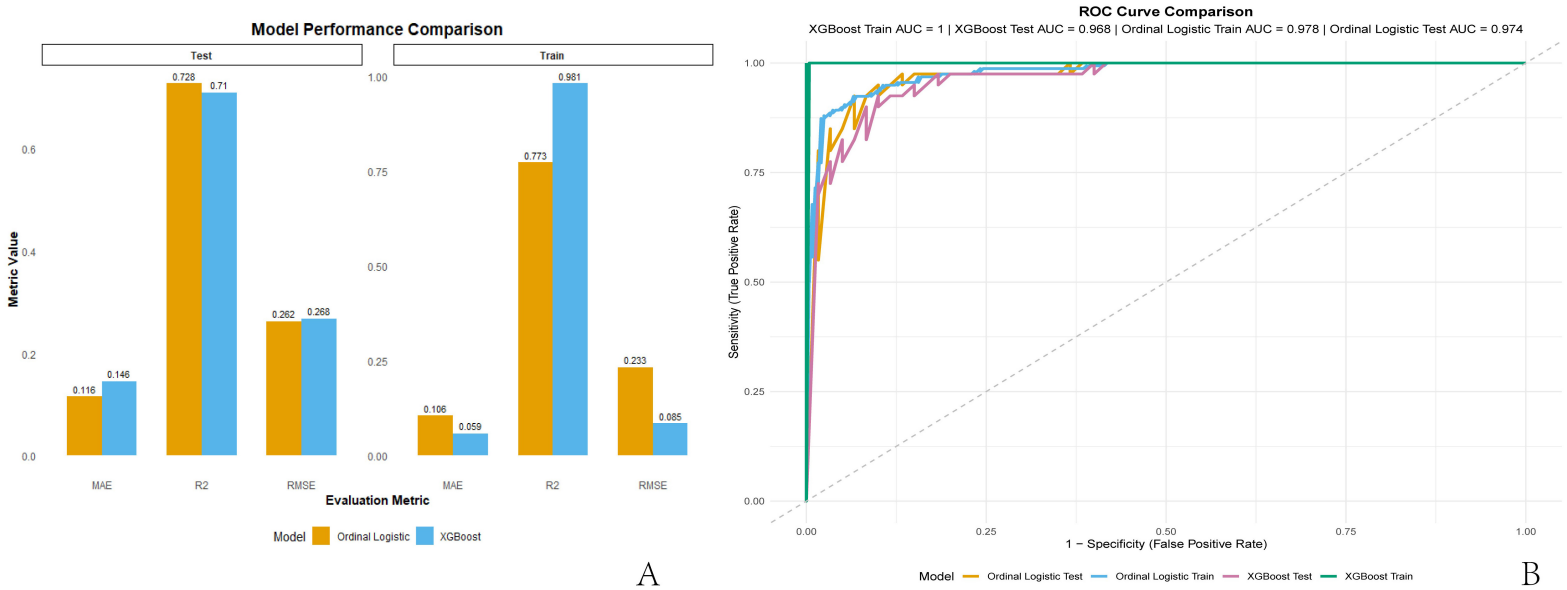
Comparison of predictive performance between the two models. **(A)** Comparison of RMSE, R^2^, and MAE in the training and testing sets; **(B)** comparison of ROC curves in the training and testing sets. *R*^2^, coefficient of determination; RMSE, root mean square error; MAE, mean absolute error; ROC, receiver operating characteristic.

Model performance comparison revealed that the XGBoost model achieved perfect performance on the training set (AUC = 1.000) and excellent performance on the test set (AUC = 0.968), slightly outperforming the Ordinal Logistic regression model. However, the Ordinal Logistic regression model exhibited higher *R*^2^ values on both the training and test sets, suggesting stronger explanatory power for variance. Additionally, the Ordinal Logistic regression produced lower RMSE and MAE than the XGBoost model, indicating smaller prediction errors. See Table 3 and Figure 3.

**Table 3:** Comparison of predictive performance between the two models.

**Model**	**Dataset**	**R^2^**	**RMSE**	**MAE**	**AUC (95% CI)**
XGBoost	Train	0.981	0.085	0.059	1.000 (1.000–1.000)
XGBoost	Test	0.710	0.268	0.146	0.968 (0.940–0.968)
Ordinal logistic	Train	0.773	0.233	0.106	0.978 (0.967–0.978)
Ordinal logistic	Test	0.728	0.262	0.116	0.974 (0.948–0.974)

*R*^2^, coefficient of determination; RMSE, root mean square error; MAE, mean absolute error; AUC, area under the receiver operating characteristic curve.

### 3.5 Sensitivity analysis

A sensitivity analysis including 34 additional patients with severe postoperative complications confirmed the robustness of key predictors (BMI, operative time, multilevel surgery, and preoperative CRP), which remained statistically significant in both the ordinal logistic regression and XGBoost models (all *P* < 0.05).

Ordinal logistic regression: The effect sizes of these predictors changed by < 8% compared with the main analysis. XGBoost: The top five features in importance ranking were identical to the main analysis.

Model performance: Ordinal logistic regression: AUC = 0.781 (vs. 0.784 in main analysis); XGBoost: AUC = 0.842 (vs. 0.846 in main analysis). These findings indicate that the exclusion of patients with severe postoperative complications did not substantially alter the effect sizes or model performance, suggesting minimal selection bias.

## 4 Discussion

### 4.1 Main findings

Lumbar spinal stenosis (LSS) is a frequently occurring degenerative condition of the spine that often results in symptoms like lower back discomfort, radiating pain in the legs, and altered sensation (13, 14). TLIF is a widely used minimally invasive surgical procedure for the treatment of LSS, which aims to relieve symptoms through spinal decompression and stabilization. Despite the continuous advancements in TLIF techniques and its widespread clinical adoption, postoperative pain management remains a significant clinical challenge. Studies report that postoperative pain following TLIF occurs in ~30%−60% of patients, with 10%−15% experiencing moderate to severe pain (15). Optimizing pain control after TLIF is therefore a clinical priority.

In this study, multiple risk factors associated with postoperative pain following TLIF were identified, with significant differences observed across pain severity groups. Notably, BMI, HbA1c, albumin, globulin, RBC, WBC, PLT, NLR, CRP, operative time, intraoperative blood loss, number of surgical segments, drainage tube placement duration, and postoperative complication rate all showed statistically significant variation. Further analysis using Ordinal Logistic Regression and the XGBoost model confirmed that age, BMI, HbA1c, albumin, globulin, WBC, PLT, NLR, CRP, operative time, intraoperative blood loss, drainage duration, surgical segments, and postoperative complications were all significant risk factors for severe postoperative pain after TLIF (*P* < 0.05). These findings are consistent with previous studies and reinforce the importance of these variables in pain management.

The XGBoost model showed a slightly higher AUC in the test dataset compared to the Ordinal Logistic Regression model, suggesting superior classification ability. However, the Ordinal Logistic Regression model demonstrated better performance in terms of explained variance (*R*^2^) and prediction error (RMSE, MAE), reflecting its suitability for ordered categorical outcomes. XGBoost analysis identified intraoperative blood loss as the most important risk factor, although this should be interpreted cautiously, as it may act as a surrogate for surgical complexity rather than a direct causal factor. Overall, both models offer complementary strengths for predicting TLIF postoperative pain, allowing selection based on specific clinical objectives and dataset characteristics.

### 4.2 Comparison with existing evidence

Prior research indicates that elderly patients may have increased pain sensitivity due to age-related changes in the nervous system, as well as reduced tissue repair capacity. Chronic conditions such as diabetes and hypertension can further impair metabolic processes and exacerbate inflammatory responses, thereby intensifying pain perception (16). Patients with high BMI are at increased risk for postoperative pain due to obesity-related chronic inflammation and increased surgical complexity; elevated HbA1c levels are associated with poor glycemic control, diabetic neuropathy, and impaired wound healing, all of which are key mechanisms contributing to more severe pain (17). Low albumin and high globulin levels reflect malnutrition and chronic inflammation, both of which are significantly associated with increased postoperative pain (18).

Postoperative elevations in WBC, PLT, NLR, and CRP indicate active inflammatory response. Inflammatory mediators stimulate nociceptors, increasing pain sensitivity (19). Specifically, WBC can release various cytokines that directly induce pain. Activated PLTs secrete pro-inflammatory substances that further intensify the inflammatory process. NLR serves as a marker of inflammatory activity, with higher values often associated with more severe postoperative pain. CRP contributes to pain by activating the complement system and facilitating the release of additional inflammatory mediators, thus amplifying both inflammation and pain perception.

Additionally, longer operative time and greater intraoperative blood loss are major contributors to postoperative pain, likely due to tissue hypoxia and intensified inflammation. In TLIF, surgeons routinely achieve hemostasis promptly when bleeding occurs, as maintaining an optimal operative view is critical. Therefore, the relationship between higher intraoperative blood loss and greater postoperative pain may reflect underlying surgical complexity rather than inadequate hemostatic technique. A key reason for elevated blood loss is often extensive paraspinal muscle dissection, which necessitates frequent use of monopolar or bipolar electrocautery (20). Greater tissue trauma often necessitates prolonged drainage, which may serve as a marker of surgical invasiveness rather than a direct cause of pain. Postoperative complications, including infection or hematoma, further exacerbate pain due to tissue damage and inflammatory processes (21).

Given these multifactorial influences on postoperative pain, advanced predictive modeling techniques are essential to accurately identify key risk factors. For instance, the XGBoost model is particularly well-suited to scenarios where the dataset includes numerous variables, complex interactions, or non-linear effects, such as predicting postoperative complications in patients with heterogeneous comorbid profiles or surgical variables (22), while Ordinal Logistic Regression provides interpretability for ordinal outcomes such as pain scores (23). Notably, the XGBoost analysis identified intraoperative blood loss as the most important risk factor for postoperative pain severity, highlighting the critical importance of intraoperative hemostasis. Clinicians should prioritize minimizing intraoperative bleeding to reduce the risk of postoperative pain. Overall, these findings align with existing literature, reinforcing the significance of perioperative factors and inflammatory markers in shaping postoperative pain outcomes.

### 4.3 Implications for clinical practice and research

The integrated findings from both the Ordinal Logistic and XGBoost models highlight several implications for clinical practice and future research. Elderly patients, those with elevated BMI, and patients with diabetes require heightened perioperative attention due to physiological decline and comorbidities. Preoperative optimization, including nutritional interventions such as a high-protein diet and correction of electrolyte imbalances, may strengthen immune function and facilitate recovery. Similarly, strict glycemic control is essential; preoperative HbA1c levels should be closely monitored to guide individualized interventions and minimize diabetes-related complications. Intraoperatively, precise surgical techniques, meticulous hemostasis, and appropriate anesthesia management remain fundamental to reducing blood loss and alleviating subsequent pain.

Postoperatively, timely drainage removal can prevent mechanical tissue stimulation, while multimodal analgesia (e.g., local anesthetics, patient-controlled analgesia, transdermal patches) provides effective pain relief. Adjustment of analgesic regimens based on inflammatory marker trends, including the incorporation of anti-inflammatory therapy, further enhances recovery (24). In addition, psychological support in the form of counseling, reassurance, and family education plays an important role in improving patients' pain tolerance and overall rehabilitation. Early mobilization is equally critical; active encouragement from nursing staff can promote circulation, strengthen muscles, and improve psychological well-being, while also preventing complications such as deconditioning, venous thromboembolism, and persistent chronic pain. Recent studies confirm that structured early mobilization programs after spine surgery can significantly reduce complications and improve long-term functional outcomes (25, 26).

From a research perspective, these findings underscore the importance of incorporating machine learning methods, such as XGBoost, alongside conventional statistical models to identify high-risk subgroups and predict postoperative outcomes with greater precision. Future prospective and multicenter studies are warranted to validate these strategies, refine individualized pain management protocols, and explore the integration of multimodal clinical and psychosocial interventions into standard perioperative care pathways.

### 4.4 Strengths and limitations

This study has several strengths. First, it comprehensively analyzed a wide range of clinical, laboratory, and surgical variables to identify risk factors for postoperative pain following TLIF, providing a holistic view of the contributing factors. Second, using both Ordinal Logistic Regression and XGBoost allowed robust comparison and validation of predictive performance. Third, the identification of intraoperative blood loss as a key modifiable risk factor offers practical clinical implications for improving postoperative pain management.

Although this study provides meaningful insights, it is not without limitations. Primarily, as a retrospective analysis, it carries the potential risk of selection bias and inaccuracies in the recorded data. Second, the number of variables analyzed was limited due to data availability, and other unmeasured factors may also influence postoperative pain. Finally, external validation of the models is necessary in independent cohorts to confirm their generalizability.

## 5 Conclusion

Postoperative pain after TLIF is influenced by multiple factors. Both Ordinal Logistic Regression and XGBoost models demonstrated good predictive ability, with intraoperative blood loss emerging as the most critical risk factor. Personalized perioperative and nursing strategies informed by these findings may help optimize postoperative pain management and improve patient recovery outcomes.

## Data Availability

The original contributions presented in the study are included in the article/supplementary material, further inquiries can be directed to the corresponding author.
