# Supplementary material for: Risk factors and nursing strategies for postoperative pain management in patients with lumbar spinal stenosis undergoing transforaminal lumbar interbody fusion: a retrospective study
Source: Front Neurol. 2025 Sep 12;16:1646333. doi: 10.3389/fneur.2025.1646333 (PMC12463963; doi:10.3389/fneur.2025.1646333)
Supplement: Supplementary file 1 [file Table_1.docx]

# Supplementary Material 1 – STROBE Checklist for Reporting Observational Studies

| Item No. | STROBE checklist item | Reported on page / section |
| --- | --- | --- |
| 1(a) | Indicate the study’s design with a commonly used term in the title or the abstract | Title and Abstract (explicitly mentions "retrospective observational study") |
| 1(b) | Provide in the abstract an informative and balanced summary of what was done and what was found | Abstract (outlines study objectives, methods, key results, and conclusions) |
| 2 | Explain the scientific background and rationale for the investigation being reported | Introduction (Section 1; elaborates on clinical significance of lumbar spinal stenosis and postoperative pain management after TLIF) |
| 3 | State specific objectives, including any prespecified hypotheses | Introduction (last paragraph; clarifies aim to identify risk factors for postoperative pain after TLIF and propose nursing strategies, with relevant hypotheses) |
| 4 | Present key elements of study design early in the paper | Abstract and 2.1 Study population (clearly states retrospective observational design) |
| 5 | Describe the setting, locations, and relevant dates, including periods of recruitment, exposure, follow-up, and data collection | 2.1 Study population (The Second Hospital of Tangshan; recruitment: Jan 2020–Dec 2023; follow-up during hospital stay) |
| 6(a) | Give the eligibility criteria, and the sources and methods of selection of participants. Describe methods of follow-up | 2.1 Study population (inclusion/exclusion criteria; data from EMR and surgical database; follow-up during hospitalization) |
| 6(b) | For matched studies, give matching criteria and number of exposed and unexposed | Not applicable (not a matched study) |
| 7 | Clearly define all outcomes, exposures, predictors, potential confounders, and effect modifiers. Give diagnostic criteria, if applicable | 2.2 Data collection (defines VAS score as outcome; predictors include age, BMI, serological indicators, surgical variables) |
| 8 | For each variable of interest, give sources of data and details of methods of assessment (measurement). Describe comparability of assessment methods if there is more than one group | 2.2 Data collection (all data from EMR; VAS pain scale; laboratory measures by standard hospital protocols) |
| 9 | Describe any efforts to address potential sources of bias | 2.2 Data collection (data extracted independently by two researchers; discrepancies resolved by consensus; 2.5 Sensitivity analysis performed) |
| 10 | Explain how the study size was arrived at | 2.1 Study population (sample size based on effect size, α=0.05, power 0.8; final n=502) |
| 11 | Explain how quantitative variables were handled in the analyses. If applicable, describe which groupings were chosen, and why | 2.4 Machine learning model (continuous variables standardized; categorical variables one-hot encoded; pain categorized as mild/moderate/severe per VAS) |
| 12(a) | Describe all statistical methods, including those used to control for confounding | 2.6 Statistical analysis (ordinal logistic regression and XGBoost; variance inflation factor to assess multicollinearity) |
| 12(b) | Describe any methods used to examine subgroups and interactions | Not applicable (no subgroup/interaction analyses performed) |
| 12(c) | Explain how missing data were addressed | 2.1 Study population and 2.6 Statistical analysis (cases with missing key variables excluded; missing data rate <5%) |
| 12(d) | If applicable, explain how loss to follow-up was addressed | Not applicable (retrospective design; only patients with complete follow-up data included) |
| 12(e) | Describe any sensitivity analyses | 2.5 Sensitivity analysis (reincluded cases with severe complications; results consistent with main analysis) |
| 13(a) | Report the numbers of individuals at each stage of study—e.g., numbers potentially eligible, examined for eligibility, confirmed eligible, included in the study, completing follow-up, and analysed | 2.1 Study population (n=502 included; see Supplementary Figure S1 flow diagram) |
| 13(b) | Give reasons for non-participation at each stage | 2.1 Study population (see flow diagram; exclusions due to incomplete data, revision surgery, or missing follow-up) |
| 13(c) | Consider use of a flow diagram | Supplementary Figure S1 provided |
| 14(a) | Give characteristics of study participants (e.g., demographic, clinical, social) and information on exposures and potential confounders | 3.1 Comparison of baseline characteristics (Table 1) |
| 14(b) | Indicate number of participants with missing data for each variable of interest | No missing data after applying exclusion criteria |
| 14(c) | Summarise follow-up time (e.g., average and total amount) | 2.2 Data collection (pain assessed at postoperative day 3; complications monitored until discharge) |
| 15 | Report numbers of outcome events or summary measures over time | 3.1 (postoperative complication rates); follow-up limited to in-hospital period |
| 16(a) | Give unadjusted estimates and, if applicable, confounder-adjusted estimates and their precision (e.g., 95% confidence interval). Make clear which confounders were adjusted for and why they were included | 3.2 (Table 2 adjusted OR, 95% CI); 3.4 (AUC, RMSE, MAE) |
| 16(b) | Report category boundaries when continuous variables were categorized | 2.2 Data collection (VAS: 0–3 mild, 4–6 moderate, 7–10 severe) |
| 16(c) | If relevant, consider translating estimates of relative risk into absolute risk for a meaningful time period | Not applicable (ordinal outcome; absolute risk not calculated) |
| 17 | Report other analyses done—e.g., analyses of subgroups and interactions, and sensitivity analyses | 3.5 Sensitivity analysis (results consistent with main analysis) |
| 18 | Summarise key results with reference to study objectives | 4.1 Main findings |
| 19 | Discuss limitations of the study, taking into account sources of potential bias or imprecision. Discuss both direction and magnitude of any potential bias | 4.4 Strengths and limitations |
| 20 | Give a cautious overall interpretation of results considering objectives, limitations, multiplicity of analyses, results from similar studies, and other relevant evidence | 4.4 Strengths and limitations; Conclusion |
| 21 | Discuss the generalisability (external validity) of the study results | 4.4 Strengths and limitations (results generalisable to similar hospital settings; caution in extrapolation to other populations) |
| 22 | Give the source of funding and the role of the funders for the present study and, if applicable, for the original study on which the present article is based | Funding section (no specific grant; “This study received no specific grant from any funding agency in the public, commercial, or not-for-profit sectors.”) |
